# Default Mode Network, Motor Network, Dorsal and Ventral Basal Ganglia Networks in the Rat Brain: Comparison to Human Networks Using Resting State-fMRI

**DOI:** 10.1371/journal.pone.0120345

**Published:** 2015-03-19

**Authors:** Adam Sierakowiak, Cyril Monnot, Sahar Nikkhou Aski, Martin Uppman, Tie-Qiang Li, Peter Damberg, Stefan Brené

**Affiliations:** 1 Department of Neurobiology, Care Sciences and Society (NVS), Karolinska Institutet, Stockholm, Sweden; 2 Department of Clinical Neuroscience, Karolinska Institutet, Stockholm, Sweden; 3 Department of Clinical Science, Intervention and Technology (CLINTEC), Karolinska Institutet, Stockholm, Sweden; Italian Institute of Technology, ITALY

## Abstract

Rodent models are developed to enhance understanding of the underlying biology of different brain disorders. However, before interpreting findings from animal models in a translational aspect to understand human disease, a fundamental step is to first have knowledge of similarities and differences of the biological systems studied. In this study, we analyzed and verified four known networks termed: default mode network, motor network, dorsal basal ganglia network, and ventral basal ganglia network using resting state functional MRI (rsfMRI) in humans and rats. Our work supports the notion that humans and rats have common robust resting state brain networks and that rsfMRI can be used as a translational tool when validating animal models of brain disorders. In the future, rsfMRI may be used, in addition to short-term interventions, to characterize longitudinal effects on functional brain networks after long-term intervention in humans and rats.

## Introduction

Many rodent models of brain disorders and disease exist [1–4], which highlights the need and possibilities of robust experimental methods that are available in both humans and rats. These methods can be used in studies that aim at understanding biological systems in human disease [5]. Resting State Functional Magnetic Resonance Imaging (rsfMRI) is used to detect functionally linked brain regions that show a synchronized pattern of spontaneous fluctuation in Blood Oxygen Level Dependent (BOLD) contrast, when the subject is in a state of rest, i.e. in the absence of a task or stimulus paradigm [6].

In humans, several networks with synchronized activity patterns exist and one example is the “Default Mode Network” (DMN) [7–9]. The DMN was first characterized by Raichle and co-workers [10] who showed that brain oxygen uptake, using Positron Emission Tomography and ^15^O-labeled radiopharmaceuticals, was overlapping with the fMRI BOLD contrast in healthy human volunteers, when they were quiet, resting, and asked to not think about anything specific. This network was characterized as the basal network of activity since it was observed that many goal-oriented tasks deactivated this network.

A similar network has also been observed in awake and anaesthetized rats, as well as in genetically modified mice [11–14].

Other robust resting state functional connectivity networks that are observed in humans as well as in rodents, including a genetically modified rat strain, are the motor/somatosensory network [7, 15–18], and the dorsal basal ganglia network [17, 19–21].

One common method to detect resting state brain networks from data generated by the rsfMRI scan, is the Seed Based Method. The method follows fluctuations of BOLD signal over time in an *a priori* chosen voxel or Volume of Interest (VOI) and finds additional voxels in the brain that are synchronized. Voxels with synchronized bold activity fluctuation with the priori chosen voxel form a defined functional brain network [22].

Analysis of DMN using rsfMRI is hypothesized to provide new insights in how the brain is affected in brain disease. Interestingly, there are several examples of experimental resting state connectivity studies showing networks that are altered in patients suffering from Alzheimer’s disease [23], dementia, depression, and Schizophrenia [24]. Although, more work is required before the methodology can be used in routine work in a clinical setting [25, 26].

The motor cortex is one of the most abundant regions affected in stroke, and animal models are being developed to mimic the pathophysiology in the disease [27].

Dopamine is a key neurotransmitter in brain reward pathways and virtually all addictive drugs induce a release of dopamine in the nucleus accumbens [28]. Pharmacological treatments of schizophrenia, ADHD, and Parkinson’s disease have in common that they via different mechanisms alter the dopamine and glutamate activity of the basal ganglia [29]. However, even though there is overwhelming data to support the key role of dopamine and brain regions such as nucleus accumbens in these brain disorders, including substance abuse, there is still little information on how the nucleus accumbens itself is affected in resting state functional connectivity studies in human and rat.

In rodents, structural brain analysis using MRI has proven valuable and protocols are developed for high resolution anatomical studies using for example diffusion tensor imaging [30]. Functional MRI studies, however, are more complex and one limitation for rodent functional MRI is that the anesthesia used during the scan is always a confounder that can impact outcome [16, 31, 32].

Recently, rsfMRI experiments were done in awake animal models to have experimental conditions more similar when comparing data from rat and human studies [19, 33]. Results indicate a minimal impact of anesthesia when non-anesthetized animals were compared to animals that were lightly anesthetized with the alpha-2 adrenergic agonist medetomidine [17].

Findings from rsfMRI on humans indicate that brain disease often is associated with changes in resting state networks. In this study, we analyzed four resting state networks in the brains of young adult human males and in young adult male rats that were under medetomidine anesthesia.

The rsfMRI data on the four networks indicate that awake humans and rats that are lightly anaesthetized have common functional brain networks.

Our results provide support for using rsfMRI as a translational tool in research on rodent models of brain disorders and disease.

## Methods

### Human Subjects

The human subjects were 13 male healthy volunteers (see S1 Table for more details) that had stated a written consent of participation with a mean age of 29.6 years (8.2 SD). All experiments were in accordance with the declaration of Helsinki, and approved by The Central Ethical Review Board of Stockholm, Sweden.

### Magnetic Resonance Imaging (MRI)—Humans

The resting-state fMRI data was acquired in a Siemens 3T whole body MR scanner (Erlangen, Germany) using a dedicated 32 channel receive only head coil. A 2D multi slice gradient echo, echo planar imaging sequence was employed with the parameters as follows; TE/TR = 36/2500 ms, time points = 180, FOV = 240 x 180 mm^2^, matrix size = 80 x 60, slices = 42, slice thickness = 3 mm, bandwidth = 2404 Hz/pixel, and parallel imaging factor = 2. During the resting-state fMRI experiment all subjects were asked to close their eyes, relax, move as little as possible and try not to think about anything specific.

### Human Data Processing

Two-pass realignment of the human resting state functional connectivity data using SPM8 software (Welcome Department of Cognitive Neurology, University College, London, UK; http://fil.ion.ucl.ac.uk.spm/) with a quality of 0.9, separation of 4mm and smoothing of 5 mm was made to correct for head motion. At the same time, the data was registered to the mean images created after the first pass.

Consecutively, segmentation of gray matter (GM), white matter (WM), and Cerebrospinal Fluid (CSF) was carried out by the SPM8 function “segment” with the parametric maps for GM, WM, and CSF provided by the toolbox. The warp frequency cutoff was 25Hz, a bias regularization cutoff of 60mm and a sampling distance of 3mm.

Subsequently, the data was normalized with a 12-parameter linear transformation to the MNI (Montreal Neurological Institute) template.

The voxel size for the images resulting from normalization was 3mm isotropic.

Gaussian smoothing kernel with FWHM of 8x8x8 mm^3 in MATLAB (MathWorks, Natick, MA) based SPM8 (SPM).

Datasets were then time-filtered by a bandpass-filter using REST toolbox (v.1.8) [34] with a range of 0.01–0.1Hz (as for the rats’ datasets, see below).

### Animal Subjects Preparation and Treatments

20 male Sprague-Dawley rats (Scanbur, Germany), 327±20 g. (10–12 weeks of age) were used. All animals had access to food and water ad libitum during the whole experiment and were subjected to a controlled 12-h light:12h dark schedule (lights on at 07:00 a.m.) Before MRI scanning the rats were initially anesthetized using 3–4% isoflurane in a 1:4 oxygen and air mixture. Animals were placed prone in an MR-compatible stereotactic holder, with the head cinched and with their teeth placed firmly in a tooth-bar with a nose-cone placed around their nose that exhausts isoflurane in a mixture of oxygen and air (ratio 1:4). To prepare for the transition to subcutaneous medetomidine (Dormitor Vet, Orion Pharma Animal Health, Sollentuna, Sweden) anesthesia during the experiment, a subcutaneous catheter was inserted while the animal was under deep isoflurane anesthesia to minimize suffering.

After insertion into the MRI scanner, the animals’ physiological conditions including body temperature, pulse (with a pulseoximeter) and respiration rate, were monitored (SA Instruments, Stony Brook, NY, USA). The core body temperature was controlled to 37 using a feedback controlled warm air system (SA Instruments, Stony Brook, NY).

Upon stabilization of the body temperature at 37°, animals received a 0.05mg/kg s.c. bolus of medetomidine subcutaneously through an infusion pump and 5 minutes after isoflurane supplement was discontinued. After 10 additional minutes, animals were supplied with a continuous flow of 0.1mg s.c./kg/h medetomidine throughout the experiment. All experiments were approved by the Ethical Committee on Animal Experiments, Stockholm North, Sweden.

### Magnetic Resonance Imaging (MRI)—Animals

The rats were scanned twice during the same session in a 9.4 T MR-scanner (VnmrJ software 3.1, Agilent, Yarnton, UK) equipped with a gradient coil of 12 cm inner diameter and a maximum gradient strength of 600 mT/m.

For excitation and detection an actively tuned 72 mm inner diameter volume coil (Rapid biomedical GmbH, Wüuzburg-Rimpar, Germany) and an actively detuned “rat brain” 4-channel phased array coil (Rapid biomedical GmbH) were used, respectively.

Three dimensional gradient echo (GE 3D) images were obtained for the slice planning with the following parameters; 2.91 ms repetition time (TR), 1.47 ms echo time (TE), 20° flip angle, 1 average, 128 x 128 x 128 data matrix and 50 x 40 x 40 mm^3^ field of view (FOV).

### High-resolution fast spin echo sequences

Anatomic reference scans were acquired using a fast spin echo sequence. A slice package of 11 transverse slices of 1 mm thickness was carefully outlined to include where anterior commissure joins medially in the central slice, which corresponds to -0.06 mm from Bregma in the caudal-rostral direction.

The following settings were used during the procedure: 3s TR, echo train containing 8 echoes where the 4th was localized to the center of k-space, 1 average, 256 x 256 data matrix and 48 x 48 mm^2^ FOV and a slice thickness of 1 mm.

The high-resolution images were multiplied with the biased field correction ratio to compensate for the bias field.

The bias field resulting from the surface coil was compensated for by recording low resolution reference scans received from both the surface and volume coil, where the matrix was set to 64 x 64. The low-resolution data were blurred using a Gaussian weighting function and zero-filled to 256x256 points. The biased field correction ratio is the ratio between the images acquired by the surface coil and the volume coil.

### Connectivity EPI data acquisition

Single shot gradient echo EPI (Echo Planar Imaging) data were acquired using identical slice positions as the anatomic reference scans. Each second, during 5 minutes, a data volume of 11 slices of 1 mm thickness with no gap between them, with each slice covering a FOV of 32 x 32 mm^2^, was acquired. The matrix size was 64 x 64, TR/TE 1000/16.33 ms, 300 repetitions, bandwidth 2791 Hz/pixel and 8 dummy scans preceded the data accumulation in order to reach the steady-state of T1.

### Animal Data processing

Consecutively to the acquisition of the rat-brain, 3300 images (11 slices with 300 repetitions), all the volumes for the different time-points were realigned to the first volume in order to correct for eventual motion artifacts. Subsequently, mean volumes for each subject were constructed. To prevent extra-brain matter from interfering with the results, skull stripping was manually performed (for each individual rat-brain) utilizing ITK-SNAP http://www.itksnap.org [35]. These images were co-registered with the individual anatomic reference images, which resulted in realignment to the anatomical template. To normalize these realigned images, an in-house standardized model of a rat brain (an arbitrary brain chosen within the group) was used as a standard template. Normalization was performed using the SPM8 tool called “normalise: estimate and write”, which uses a 12-parameter linear transformation in order to fit a given image. Here, the anatomical volume of each individual subject was fitted to a template (in this case, the arbitrary brain chosen within the group). Gaussian smoothing, 0.6 mm, was performed with the purpose of reducing small variances, and thermal noise.

A band-pass filter with a bandwidth of 0.01–0.1 Hz was applied by the REST toolbox (v.1.8). Band-pass filtering suppresses drift and some artifacts due to cardiac rhythm, respiration, as well as thermal noise, while preserving spontaneous BOLD fluctuations.

SPM8 was used in order to perform the realignment, coregistration, normalization and smoothing.

### Seed based analysis

The volume of interest (VOI) was chosen in a relevant brain region. The Pearson correlation coefficients of BOLD-fluctuations between every voxel contained within the brain and that a priori VOI were then calculated. The seed volume was 3x3x3 mm for the human data and 0.5x0.5x1.0 mm for the animal data (i.e. one voxel in both cases). The seeds in the human data were placed with the help of “MNI<->Talairach with Brodmann Areas (1.09)” (An application with components of the Yale BioImage Suite Package: http://noodle.med.yale.edu/~papad/mni2tal/), see Table 1 for details on seed placement.

**Table 1:** Seed placement in humans for; DMN, motor network, dorsal basal ganglia network, and ventral basal ganglia network.

Network	Brain region	Coordinates in Talairach *(in mm) X; Y; Z*
DMN	Posterior Cingulate Cortex	0; -47; 29
Motor Network	Primary Motor Cortex	+/-46; -13; 38
Dorsal Basal Ganglia Network	Putamen	+/-21; 0; 7
Ventral Basal Ganglia Network	Nucleus Accumbens	+/-14; 5; -8
Somatosensory Cortex Network	Primary Sensory Cortex	+/-43; -24; 44
Put-BA6	Brodmann Area 6 cluster of the Putamen network	1; 11; 49
DMN-PFC	The Pre Frontal Cortex cluster of the Default Mode Network	0; 55; 5
NAcc-BA10	Brodmann Area 10 cluster of the Ventral Basal Ganglia Network	1; 47; -13

The coordinates of the seeds in the animal data, which all are placed in both brain hemispheres, were carefully placed with the help of Paxinos rat atlas [36] to match the morphological structures as much as possible (see Table 2 and S1 Fig. for coordinates and seed placements).

**Table 2:** Seed placement in rat for; DMN, motor network, dorsal basal ganglia network, and ventral basal ganglia network.

Network	Brain region	Coordinates in Bregma *(in mm) M-L; D-V; A-P*
DMN	Rostral Anterior Cingulate Cortex	+/-0.5; 2.5; 1.7
Motor Network	Primary Motor Cortex	+/-3.0; 1.5; 1.7
Dorsal Basal Ganglia Network	Caudate Putamen	+/-3.5; 4; -0.3
Ventral Basal Ganglia Network	Nucleus Accumbens	+/-1.5; 6.5; 1.7
Somatosensory Cortex Network	S1	+/-5; 2; -0.8
DMN Rostral	Prelimbic Cortex	0; 3.5; 2.5
DMN Caudal	Cingulate Cortex, area 1	0; 1.5; 0

The WM and CSF signals in the human data were used as regressors during the seed based analysis for the human data.

The signals from the CSF and the white matter in the animals were extracted from manually segmented masks of the EPI volumes, and used as regressors in the analysis (see S2 Fig. and S3 Fig.) for masks displayed on anatomical reference images).

The REST toolbox was used to perform the Seed-based analysis for both humans and animals, with the seed placed bilaterally (apart from the DMN in humans). In addition, unilateral seeds were also analyzed to confirm the bilateral extension of the three human networks and for all rat networks as well as the somatosensory network in human and rat, which are described in the supplementary section (see S4 Fig. and S5 Fig.).

### Statistical analysis: One Sample T-test

In order to evaluate the results from the Seed-based approach, of humans as well as rats, and the similarity between them, we used a One-Sample T-test on the Fisher z-transformed data obtained from the seed-based approach. The statistical significance was assessed with t-score threshold corresponding to a voxel-wise p-threshold p<0.0005 without correcting for multiple comparison.

The voxel size after pre-processing was estimated (with the AFNI program 3dFWHMx) to be 12.33 mm^3^ for the human data and 1.5 mm^3^ for the rat data (averaged for the 4 networks for each species individually, calculated by SPM12). To calculate a minimum cluster size for the human data and the rat data, probability simulations based on *AlphaSim*, using 10^5^ iterations indicate that the probability of random field of noise producing a cluster of size ≥130 is at p<0.0005 after the noise was thresholded at pixel level with p<0.0005 for humans and a cluster of size ≥33 for rats at the same p-value.

I.e. the final statistical significance was improved by also enforcing a minimum voxel cluster size of 130 contiguous voxels in the human data and 33 voxels in the rat data in SPM8. After specifying the original and final voxel sizes, as well as the uncorrected threshold value, the AFNI [37] program, AlphaSim, was used to compute a list of probabilities corresponding to different cluster sizes produced by a random field of noise.

This step was performed using SPM8. In this study, the figures display the Functional Correlation values (FC-values) in the areas where the corresponding p-value for the T-score maps is less than 0.0005 uncorrected and minimum cluster corrected. These images were constructed with ImageJ [38], SPM8, SPM12 (Welcome Department of Cognitive Neurology, University College, London, UK; http://fil.ion.ucl.ac.uk.spm/) and the REST-toolbox.

### Correlation Matrices

Moreover, two matrices (one for humans and one for rats) were calculated by averaging correlation scores, Fisher z-transformed, obtained by single seeds, described in Table 1 for humans, and Table 2 as well as S1 Fig. for rats. The scores were calculated by the functional connectivity “ROI-wise” function in REST. All individual subject matrices were then averaged in MATLAB, and the final matrix was built by in-house written MATLAB scripts. To explore the networks further, five additional seeds placed within the networks, three in the human brain and two in the rat brain were added in the analysis. These seeds were selected in nodes that showed to be correlated and had high FC-values to the bilateral seed networks with the help of the REST toolbox “viewer” function.

All figures were adjusted for publication with Adobe Photoshop CS5.

## Results

### Default Mode Network in Human and Rat Brain

**Fig 1:**
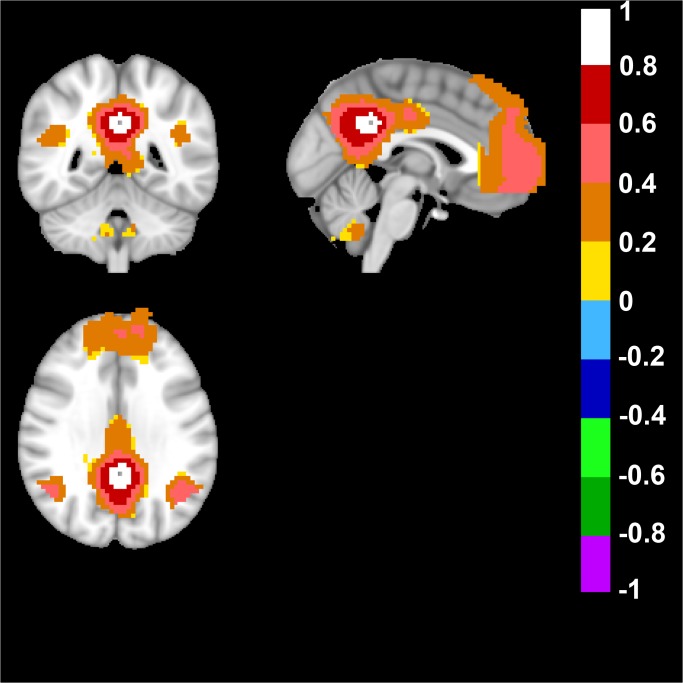
Human Default Mode Network. The default mode network (DMN) was derived from a seed placed in Posterior Cingulate Cortex (Talairach coordinates in mm: 0; -47; 29). Top left image displays an axial, top right a sagittal and bottom image a coronal view of the human brain. Note the color-coded positive FC-values of the DMN in the posterior cingulate/retrosplenial cortex (precuneus) and bilateral parietal cortex in the axial view. In the sagittal and coronal view note the anterior prefrontal cortex, bilateral parietal cortex and posterior cingulate/retrosplenial cortex (precuneus). FC-values are displayed with a pseudo-colored scale bar; with increments of 0.2 each designated a color (see the color-bar in the image).

In the Human Brain, the default mode network (DMN) was derived from the seed-based approach, when the seed was placed in Posterior Cingulate Cortex (Talairach coordinates: 0; -47; 29). As expected, and confirming previous data, a network was detected that included anterior prefrontal cortex, posterior cingulate cortex/retrosplenial cortex (precuneus), and bilateral parietal cortex (Fig. 1) [39].

**Fig 2:**
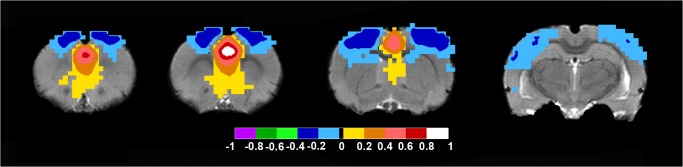
Rat Default Mode Network. When the bilateral seed was placed in the Rostral Anterior Cingulate Cortex approximately Bregma in mm: +/-0.5; 2.5; 1.7 the DMN was distinguished. Axial rat brain images are sorted from left to right in rostral to caudal order. The image to the far left shows positive color-coded FC-values in pre limbic cortex, infra limbic cortex, orbital cortex and cingulate cortex. The two middle images show positive FC-values in cingulate cortex, septal nuclei. Also, note the bilateral anticorrelated color-coded regions in the motor-sensory cortical region throughout the images. Note the lack of signal in hippocampus. FC-values are displayed with a pseudo-colored scale bar; with increments of 0.2 each designated a color (see the color-bar in the image).

When the seed in the rat brain was placed in the Rostral Anterior Cingulate Cortex approximately Bregma in mm: +/-0.5; 2.5; 1.7 the DMN was distinguished, which included retrosplenial cortex, pre limbic cortex, infra limbic cortex, orbital cortex, the cingulate cortex, septal nuclei, thalamic nuclei and a bilateral anticorrelated motor-sensory region (Fig. 2 and S6 Fig. for full anatomical coverage).

### Motor Network in Human and Rat Brain

**Fig 3:**
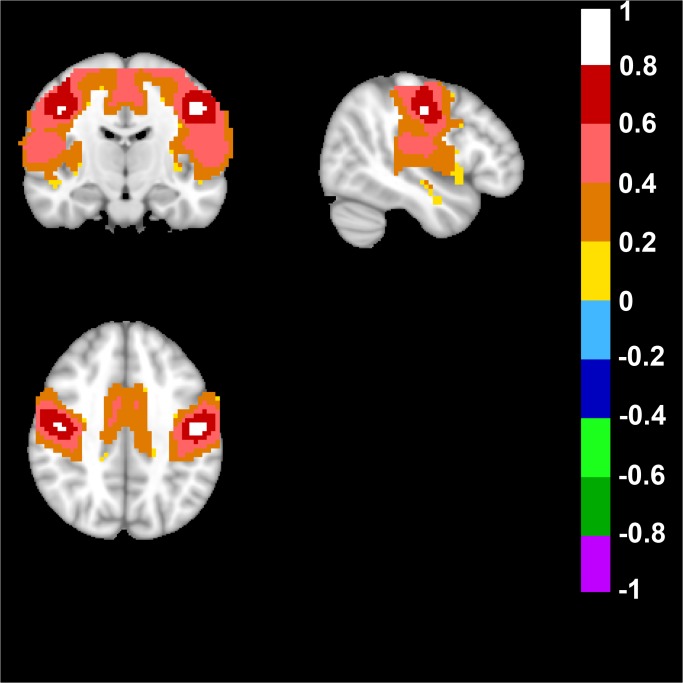
Human Motor Network. This network was derived from a bilateral seed placed in the Primary Motor Cortex region (Talairach coordinates in mm: +/-46; -13; 38). Top left image displays an axial, top right a sagittal and bottom image a coronal view of the human brain. Note the possitive FC-values of the primary motor cortex, somatosensory cortex and the supplementary motor area, the unilateral sagittal view of the motor cortex as well as the somatosensory cortex, and the coronal bilateral view of the primary motor cortex and the supplementary motor area. FC-values are displayed with a pseudo-colored scale bar; with increments of 0.2 each designated a color (see the color-bar in the image).

Next, the motor network was analyzed in the human brain, which was derived from a seed placed in the primary motor cortex (Talairach coordinates: +/-46; -13; 38). The network included the bilateral primary motor cortex, and a part of the bilateral primary somatosensory cortex as well as the supplementary motor area (Fig. 3).

**Fig 4:**
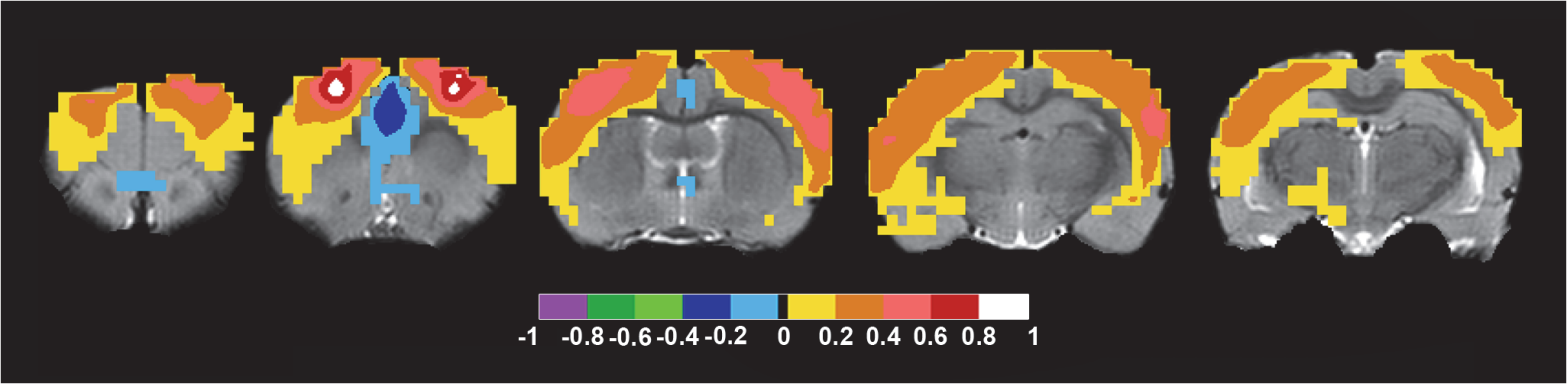
Rat Motor Network. To define the motor-sensory network in the rat brain we placed a bilateral seed in the corresponding part of the brain in the primary motor cortex, approximately Bregma in mm: +/-3.0; 1.5; 1.7. Axial rat brain images are sorted from left to right in rostral to caudal order. The image to the far left shows bilateral positive low FC-values in M1 region and negative correlation values in medial orbital cortex. The second image to the left shows bilateral positive FC-values in M1 as well as S1 and negative FC-values in cingulate gyrus and orbital cortex. The two images to the right show bilateral positive FC-values in M1, S1, and S2. FC-values are displayed with a pseudo-colored scale bar; with increments of 0.2 each designated a color (see the color-bar in the image).

To detect the motor network in the rat brain a seed was placed in the corresponding primary motor cortex (M1), approximately Bregma in mm: +/-3.0; 1.5; 1.7. Interestingly a network including the M1-region and parts of the somatosensory cortex area 1 (S1) and area 2 (S2), was detected. Also, an anticorrelated network was detected in the medial orbital cortex, ventral orbital cortex, cingulate gyrus (Fig. 4 and S7 Fig. for full anatomical coverage).

Apart from seeds in the motor cortex, somatosensory cortex was also explored in relation to the motor cortex. The results of the networks extent (in humans and in rats), as well as the overlap with the motor cortex, can be found in S8, S9, S10, S11, and S12 Figs.

### Dorsal Basal Ganglia Network (Caudate and Putamen) in Human and Rat Brain

**Fig 5:**
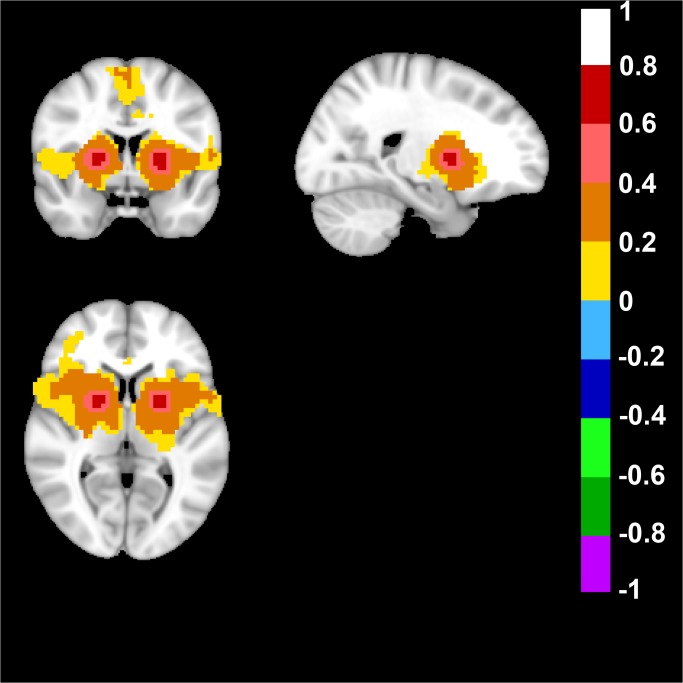
Human Dorsal Basal Ganglia Network. A dorsal basal ganglia network in the human brain was defined by placing a bilateral seed in Talairach coordinates in mm: +/-21; 0; 7. Top left image displays an axial, top right a sagittal and bottom image a coronal view of the human brain. Note the possitive FC-values of the bilateral putamen, as well as the supplementary motor area in the axial view, the unilateral sagittal view of putamen, and the coronal bilateral view of putamen. FC-values are displayed with a pseudo-colored scale bar; with increments of 0.2 each designated a color (see the color-bar in the image).

We also analyzed a dorsal basal ganglia network in the human brain and this network was defined by placing a seed in Talairach coordinates: +/-21; 0; 7 and the resulting network included bilateral Putamen regions, and the supplementary motor area (Fig. 5).

**Fig 6:**
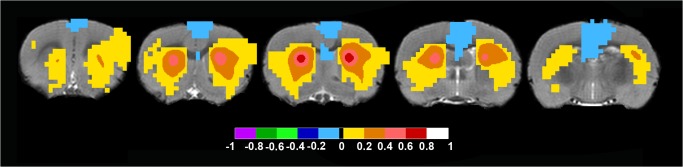
Rat Dorsal Basal Ganglia Network. To define this network, a bilateral seed was placed at approximately Bregma in mm: +/-3.5; 4; -0.3. Axial rat brain images are sorted from left to right in rostral to caudal order. The images from the far left to the right all show bilateral positive FC-values bilateral caudate putamen and week positive FC-values in the rostral somatosensory cortex in the three images to the left. Note that all images also includes an anticorrelated caudal cingulate gyrus region. FC-values are displayed with a pseudo-colored scale bar; with increments of 0.2 each designated a color (see the color-bar in the image).

The corresponding rat brain seed was placed in the Caudate Putamen at approximately Bregma in mm: +/-3.5; 4; -0.3 and the analysis gave a network including bilateral dorsal caudate putamen, rostral somatosensory cortex and an anticorrelated cingulate gyrus region (Fig. 6 and S13 Fig. for full anatomical coverage).

### Ventral Basal Ganglia Network in Human and Rat Brain

**Fig 7:**
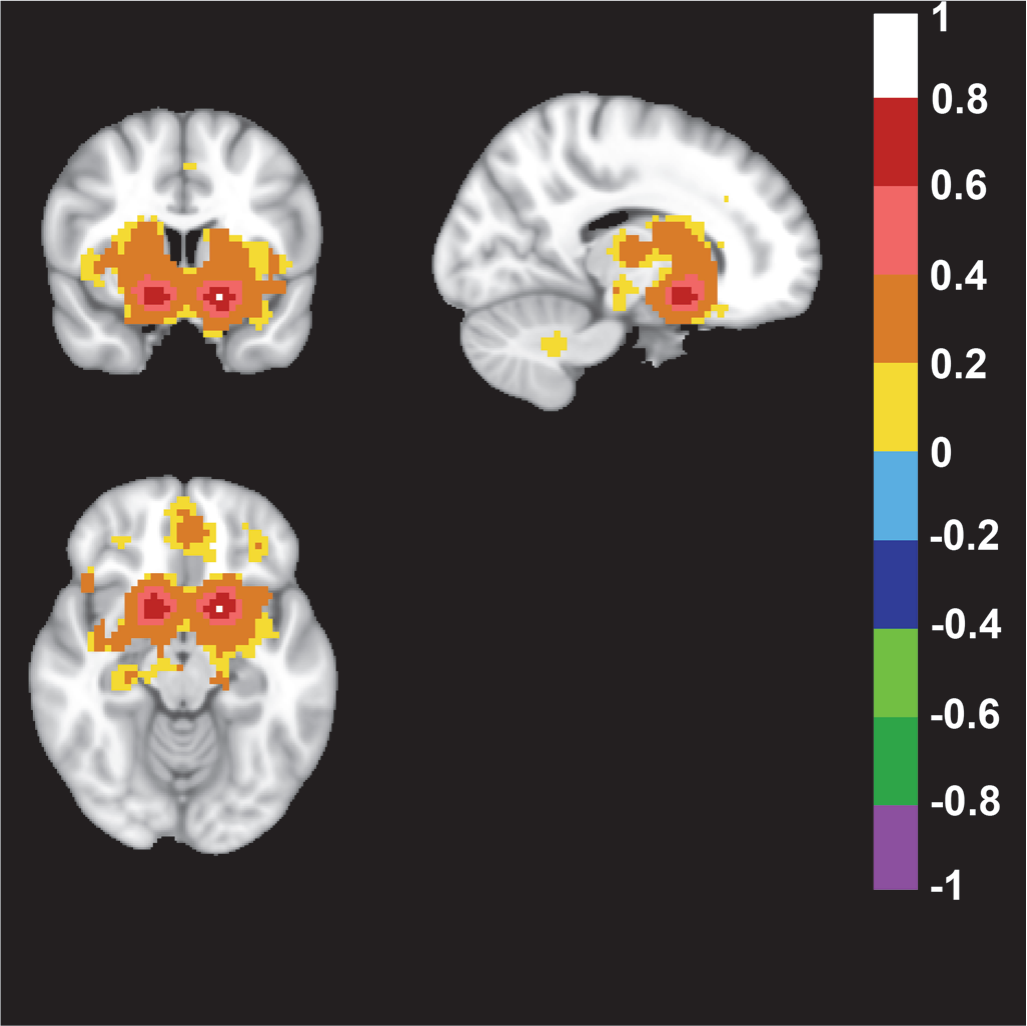
Human Ventral Basal Ganglia Network. A bilateral seed placed in Talairach coordinates in mm: +/-14; 5; -8 revealed the ventral basal ganglia network. Top left image displays an axial, top right a sagital and bottom image a coronal view of the human brain. Note the possitive FC-values of the bilateral nucleus accumbens in the axial view, the unilateral sagittal view of nucleus accumbens and the possitive FC-values in the caudate as well as putamen, and the coronal bilateral view of nucleus accumbens as well as lower possitive FC-values indicating a part of the ventromedial prefrontal cortical region. FC-values are displayed with a pseudo-colored scale bar; with increments of 0.2 each designated a color (see the color-bar in the image).

A seed was placed in Talairach coordinates: +/-14; 5; -8 to target a ventral basal ganglia network in the human brain. The analysis generated a network that included nucleus accumbens, caudate, putamen, and the ventromedial prefrontal cortex (Fig. 7).

**Fig 8:**
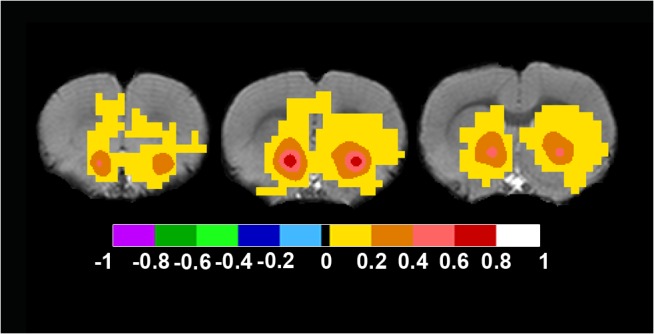
Rat Ventral Basal Ganglia Network. This network was detected by placing the bilateral seed in approximately Bregma in mm: +/-1.5; 6.5; 1.7. Axial rat brain images are sorted from left to right in rostral to caudal order. The image to the far left shows bilateral lower positive FC-values in prefrontal cortex, and bilateral higher positive FC-values in the nucleus accumbens. The middle image shows bilateral positive FC-values in nucleus accumbens, and lower positive FC-values in medial caudate putamen as well as prefrontal cortex. The image to the far right shows positive FC-values in nucleus accumbens and lower positive FC-values in caudate putamen. FC-values are displayed with a pseudo-colored scale bar; with increments of 0.2 each designated a color (see the color-bar in the image).

Seed based analysis of rat brain data, when the seed was placed in approximately Bregma in mm: +/-1.5; 6.5; 2.7, gave a network that included nucleus accumbens as well as a part of the medial caudate putamen, dorsal caudate putamen, and a part of prefrontal cortex (Fig. 8 and S14 Fig. for full anatomical coverage).

### The Human and Rat Correlation Matrices

**Fig 9:**
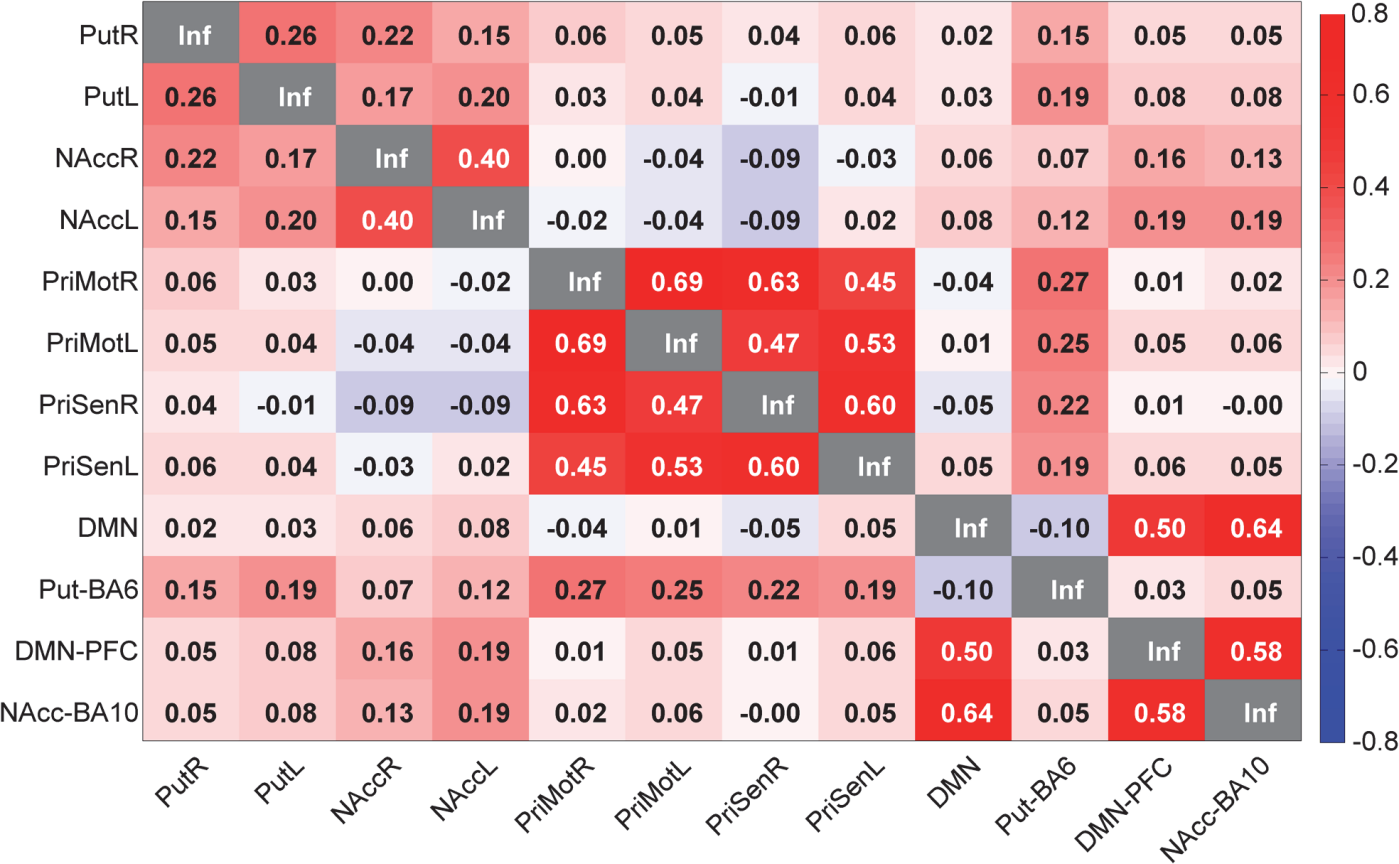
Human Seed Correlation Matrix. Note the high correlations between the somatosensory and motor regions as well as the high correlations between the prefrontal regions of the brain. Put = Putamen, NAcc = Nucleus Accumbens, PriMot = Primary Motor Cortex, PriSen = Primary Sensory Cortex, DMN = Posterior Cingulate Cortex, Put-BA6 = Brodmann Area 6 within the Putamen network DMN-PFC = Prefrontal Cortex within the DMN, NAcc-BA10 = Brodmann Area 10 within the ventral basal ganglia network. The values displayed are Fisher z-transformed correlation values.

**Fig 10:**
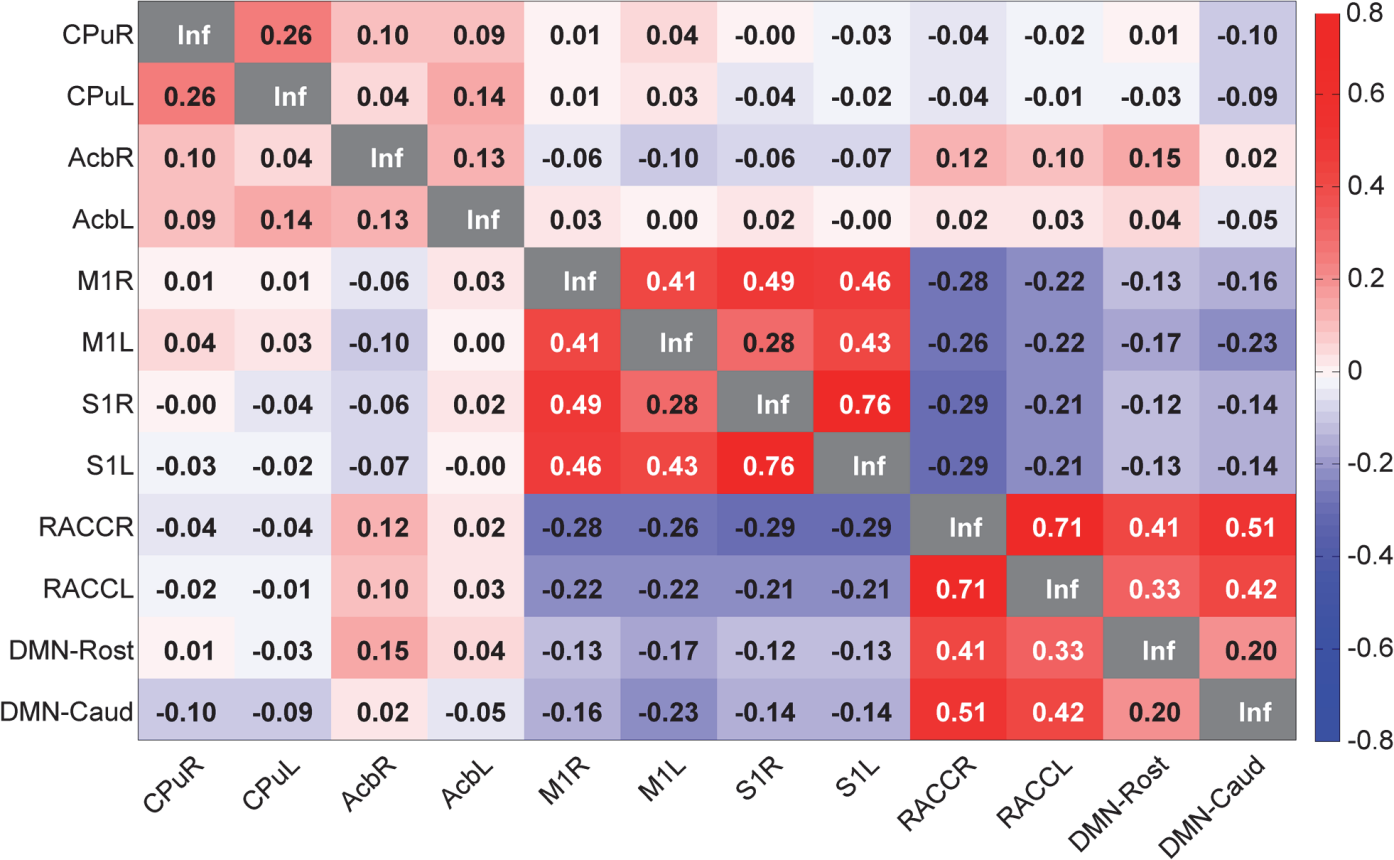
Rat Seed Correlation Matrix. Note the high correlations between the somatosensory and motor regions as well as the high correlations between the seeds in the DMN. CPu = Caudatus Putamen, Acb = n. Accumbens, M1 = primary motor cortex, S1 = primary somatosensory cortex, RACC = Rostral Anterior Cingulate Cortex, DMN-Rost = PrL: Prelimbic Cortex DMN-Caud = Cg1: Cingulate Cortex, area 1. The values displayed are Fisher z-transformed correlation values.

Human and rat correlation matrices were calculated, and most often there were striking similarities between the matrices and the seed correlation scores when comparing species (Fig. 9 and Fig. 10). One example, of similarity from the cross-species comparison (between humans and rats), was the bilateral dorsal basal ganglia seed, which had the same value (0.26, in Fisher transformation of correlation score) in human (PutR vs. PutL, Fig. 9) and in rat (CPuR vs. CPuL, Fig. 10). However, there were exceptions, and in the ventral basal ganglia bilateral seed comparison, the bilateral seed correlation in the nucleus accumbens had a higher correlation value in humans (0.4 in NAccR vs. NAccL) than in rats (0.13 in AcbR vs. AcbL). The motor and somatosensory regions in both species had high bilateral within motor and somatosensory region correlation scores as well as high correlation scores between motor regions and somatosensory regions. For the within network analysis of the DMN in both human and rat, the correlation between the different DMN nodes was similar (Fig. 9 for humans and Fig. 10 for rats).

When considering the between network correlations (in Fig. 9 and Fig. 10), it was apparent that the dorsal basal ganglia (PutR and PutL in humans as well as CPuR and CPuL in rats) network seeds and the ventral basal ganglia network seeds (NAccR and NAccL in humans as well as AcbR and AcbL in rats) were not highly correlated in either species. Nor was the dorsal basal ganglia highly correlated with any other networks or network nodes. The motor and somatosensory network seeds (PriMotR, PriMotL, PriSenR, and PriSenL) had a correlation value of 0.19–0.27 when correlated to the supplementary motor area in the Putamen network (Put-BA6) in humans, and the corresponding seeds (M1R, M1L, S1R, and S1L) were anti correlated to the DMN as well as network nodes (RACCR, RACCL, DMN-Rost, and DMN-Caud) in rats. Additionally, the DMN seed (DMN) and the prefrontal node of the ventral basal ganglia network (NAcc-BA10) were highly correlated (0.64) in the human brain.

Concerning the within network correlations (in Fig. 9), in humans, it was evident that the correlation within the dorsal basal ganglia network (PutR and PutL) and the second node (Put-BA6) was week (0.15–0.19). Similarly, the ventral basal ganglia network seeds (NAccR and NAccL) and the second node (NAcc-BA10) were also weekly correlated (0.13–0.19). Analysis of DMN and the ventromedial prefrontal cortex node (DMN-PFC) indicated a high correlation (0.5).

In rats (Fig. 10), the two DMN nodes (DMN-Rost and DMN-Caud) were anti-correlated (-0.12–-0.23) to the motor and somatosensory seeds (M1R, M1L, S1R, and S1L). The DMN seeds (RACCR and RACCL) were highly correlated (0.33–0.51) to the two other DMN network nodes (DMN-Rost and DMN-Caud).

## Discussion

In recent years functional resting state connectivity networks in the human brain have been visualized with MRI. In experimental studies, the technique has the potential to separate patients suffering from Alzheimer, depression, Schizophrenia from matched control subjects [24], however more development is required prior to routine clinical application [25, 26]. In this study, we aimed to characterize functional resting state networks that are present in both the human and rat brain.

To harmonize protocols to be used for translational studies when comparing networks in human and rodent brains we used the same statistical software, calculations, and hypothesis when placing seeds in order to detect DMN and other networks in the human and rat brain.

Obviously there are large differences in the structure and function of the human and rodent brain, however the basic neurotransmitter systems and structural connections and projections are the same [40]. Our visualization of the DMN using a seed based analysis in the human brain is in accordance with data presented by others [8, 10]. The rat DMN included prefrontal cortical regions, cingulate cortex, and retrospineal cortex, which was similar to the human DMN. However, the septal nuclei, thalamic nuclei and bilateral motor sensory regions were included in the rat but not in the human. Thus, our analysis revealed brain regions that were present in both species but also examples of regions that only were included in the DMN in either human or rat.

Recently, Lu and co-workers defined the DMN in the rodent brain using the exploratory approach of Independent Component Analysis (ICA) [11]. However, the current study used the seed based methodology on the rodent brain to reveal the DMN. Using the approach in this study, infralimbic cortex and prelimbic cortex were included, in the rat DMN, in addition to retrosplenial cortex, orbital cortex, cingulate cortex, septal nuclei, thalamic nuclei and a bilateral anticorrelated motor-sensory region, which made the network more similar to findings in previous work [12, 33, 41].

In the study by Lu and co-workers, the entire DMN was distinguished in one single ICA component, where the number of components was set to either 20 or 40. This implies a strong robustness of the network [11]. Another study by Schwarz and co-workers demonstrated DMN-like networks using the Seed Based Method, and placing seeds in different locations along the anatomically defined hippocampal-prefrontal network of the rat brain. Parts of the hippocampal formation were included in the networks demonstrated while this was not the case in the current study after white matter and CSF was compensated for in the preprocessing [12]. The correlation coefficients of the DMN in the present study were also observed to be higher. It could be explained by two reasons: the signal regression of white matter and CSF as well as the anesthesia protocol.

The method used here, was to mask out the CSF and the white matter, obtain an average signal from these masks, and finally use them as regressors in the further analysis. In contrast, the paper by Schwarz et al. [12] regressed physiological noise from the time series by recording and using them as described by van Buuren et al. [42], which leads to an overall decrease of correlation coefficients.

In the study by Schwarz et al. animals were scanned 20 minutes after isoflurane was tapped to zero, and the dose of medetomidine was higher than in the present study. In the current study the data acquisition started 90 minutes after isoflurane was terminated and the animal was on a low maintenance dose of medetomidine. The apparent different experimental conditions with stronger pharmacological influence by anesthetics, already well described by Magnuson et al. [43], as well as the difference in seed placement in the Schwarz study could explain the different connectivity patterns when compared to this study.

Nevertheless, the rodent studies are conducted with anesthesia, which very well could influence and mask the activity in the brain [44]. For example, in a study on the human brain the DMN is affected by propofol anesthesia [45]. Thus it is possible that the differences in the extent of the DMN when comparing the present study to the study by Lu and co-workers [11] is caused by a number of different factors. For example, anesthesia protocols because isoflurane is maintained throughout the whole scanning time in the study by Lu and there was no maintenance dose of isoflurane in the present study as well as differences in analysis methodology (such as WM/CSF signal regression), statistical threshold, and sample size.

Another difference that was noticed when comparing human and rat DMN, was that the rat but not the human had anticorrelated motor cortex regions. This confirms previous studies with DMN-like anticorrelated networks in rats [41]. Moreover, other DMN studies on humans have demonstrated anticorrelated motor areas [46–48]. One of the possible reasons for the difference between the present study, where no anticorrelated motor cortex regions were found, and those studies is that global signal was regressed in their data preprocessing, which was not performed in the present study. However, a comparison of the two techniques to regress out white matter and cerebrospinal fluid shows that “masking” out signals from the CSF and white matter is superior compared to global signal reduction [49].

In the motor network of both species, both the bilateral primary motor cortex was detected as well as parts of the primary somatosensory cortex. The analysis showed a high similarity between the two species. Although, in the rat brain, the motor network covered a more extensive part of both the primary and the secondary somatosensory cortex.

The motor-sensory network in humans, displayed a bilateral motor network, and in the rodent brain this network was correlated to parts of the sensory cortical regions as well as anticorrelated to a rostral part of the cingulate cortex. The correlation between the rats sensory and motor regions has been described earlier. For example, co-activation of both the sensory and motor regions of the cortex is detected when an electrical stimulation is applied on an extremity [15]. Additionally, FC-values as high as 0.64–0.84 between the motor regions and the somatosensory cortical regions are noted. Even though the brains of humans and rats are different in size, the cortical structure and microstructure are similar [40]. The rat brain is smaller in size and the neurons are denser in the cortical region, whereas in humans the cortex is bigger in surface area. Interestingly, the neurons of the human cortex have more synapses per neuron compared to the rat [40].

The cortical innervation of the motor area is more compartmentalized in the human compared to the rat brain where these systems are overlapping [50]. In the rat, there are synaptic connections between the motor cortex and the somatosensory cortex [51], which link the two cortices. Thus, reciprocal response is detected when somatosensory cortex is stimulated with an electric current, and a recording electrode in the motor area as well as vice versa. Although, when the stimulus is placed in the somatosensory area, the input on motor areas is more intense than the other way around. This interlink of activation could explain why the distinction between the rat somatosensory cortex and motor cortex is more diffuse in terms of the BOLD-signal.

Since we found anticorrelated motor regions to rat DMN, the discovery of a rostral anticorrelated cingulate cortex that appeared in the bilateral seed based analysis of the motor regions was not surprising. It is in line with the fact that the corticospinal system in rats originates in the rostral cingulate cortex [50] and here we show the functional connection to the motor and somatosensory cortex.

The dorsal basal ganglia network showed bilateral maps of the Putamen in humans, and Dorsal Caudate Putamen in rats. However, it was only in the rat that Cingulate Gyrus region was anticorrelated to the network.

The dorsal basal ganglia resting state network (caudate and putamen) were well defined with the seed-based modeling in the human brain as well as in the rat brain, which is in accordance with other studies [16, 17, 20, 52]. Although, there is evidence of a link between the prefrontal structures and the dorsal caudate [20], which we also detect in the human data, it was not detected in our data set of the rat brain. However a low correlated cortical somatosensory region was detected.

The ventral basal ganglia network was found in both rats and humans and it included bilateral nucleus accumbens and parts of Putamen in humans, and dorsal caudate putamen in rats. The network also included a part of the prefrontal cortical region. No major difference of anatomical areas included in the network was found.

Nucleus Accumbens and prefrontal cortex, in both humans and in rats, were included in the same resting state network confirming previous observations from humans [53].

Another observation in the present study was the high FC-values in the rats that were in the range seen in human studies, which is different from prior rodent studies [12, 54]. The reason for this can depend on several factors. Firstly, in this study, an anesthesia protocol to minimize the influence of isoflurane was used. Secondly, masked out WM and CSF time-courses were used as regressors for the processing, leading to even higher correlations. Apart from this, small seeds were used, and as few data transformations as possible were applied to keep the signal as similar to raw data as it would allow. We can also rule out the possibility of anti-correlations due to white matter regression in the rat data, since t-maps were also generated only using the CSF masks (see S15 Fig. for the scale-bar and S16, S17, S18, S19, and S20 Figs. for the networks).

An interesting point to note is the correlation matrix similarity between both humans and rats. One overall feature was that the correlation score was slightly higher in the human matrix compared to the rat matrix. The reason for this difference can depend on methodological factors such as anesthesia in rats, and not humans, difference of preprocessing etc. The general correlation scores were very similar within and between networks. For example, the somatosensory and motor networks were strongly correlated in both species, as well as the DMN and other nodes of the DMN. In the dorsal basal ganglia network (where the seed was placed in the putamen in humans and the caudate putamen in rats) the seeds were not that highly correlated within the network in either species (0.26) but between species and it was the same correlation strength highlighting the translational nature of this network. In contrast, within network correlation (bilateral) with seeds in nucleus accumbens had stronger correlation between in human compared to the rats. It is possible that this finding could be part of an evolutionary aspect of the networks and highlight functional differences. One example of functional difference when comparing humans and rats is the typical locomotor response in rats following a challenge of for example drugs of abuse [28, 55, 56], which is suggested to primarily be mediated via nucleus accumbens

To summarize, remarkable similarities were detected within four robust, well-defined common functional networks that were observed with rsfMRI using similar methodology in humans as in rats. The results support the validity of further use of this methodology for evaluating brain networks in rat models.

## Supporting Information

S1 Fig Seed placement (rat brain). DMN-Rost = PrL; Prelimbic Cortex, RACC = Rostral Anterior Cingulate Cortex, M1 = primary motor cortex, Acb = n. Accumbens, DMN-Caud = Cg1; Cingulate Cortex area 1, CPu = Caudatus Putamen, S1 = primary somatosensory cortex.
(TIF)

S2 Fig White Matter (WM) mask (rat brain). (TIF)

S3 Fig Cerebrospinal Fluid (CSF) mask (rat brain). (TIF)

S4 Fig T-maps of unilateral seeds of the four networks including Somatosensory Cortex Network (human brain). Top left = the dorsal basal ganglia network top right = the somatosensory network bottom left = the ventral basal ganglia network bottom right = the motor network.
(TIF)

S5 Fig T-maps of unilateral seeds of the four networks including Somatosensory Cortex Network (rat brain). Top left = the DMN top right = dorsal basal ganglia network middle left = the ventral basal ganglia network middle right = the somatosensory network middle left = the motor network.
(TIF)

S6 Fig The full range Default Mode Network (rat brain). (TIF)

S7 Fig The full range Primary Motor Cortex Network (rat brain). (TIF)

S8 Fig The Primary Somatosensory Cortex Network (rat brain). (TIF)

S9 Fig The full range Primary Somatosensory Cortex Network (rat brain). (TIF)

S10 Fig The Primary Somatosensory Cortex Network (human brain). (TIF)

S11 Fig The Primary Somatosensory Cortex Network (human brain). Displayed in the primary motor cortex orthogonal view.
(TIF)

S12 Fig The Primary Motor Cortex Network (human brain). Displayed in the primary somatosensory cortex orthogonal view.
(TIF)

S13 Fig The full range Dorsal Basal Ganglia Network (rat brain). (TIF)

S14 Fig The full range Ventral Basal Ganglia Network (rat brain). (TIF)

S15 Fig Color scale of the T-values used for all T-map networks in both species. (TIF)

S16 Fig The full range Default Mode Network (rat brain) with only CSF regression (T-map). (Color scale indicates T-values)
(TIF)

S17 Fig The full range Primary Motor Cortex Network (rat brain) with only CSF regression (T-Map). (Color scale indicates T-values)
(TIF)

S18 Fig The full range Dorsal Basal Ganglia Network (rat brain) with only CSF regression (T-Map). (Color scale indicates T-values)
(TIF)

S19 Fig The full range Ventral Basal Ganglia Network (rat brain) with only CSF regression (T-Map). (Color scale indicates T-values)
(TIF)

S20 Fig The Primary Somatosensory Cortex Network (rat brain) with only CSF regression (T-Map). (Color scale indicates T-values)
(TIF)

S1 Table Human subject details. (DOCX)
