# Supplementary figures and images for: Default Mode Network, Motor Network, Dorsal and Ventral Basal Ganglia Networks in the Rat Brain: Comparison to Human Networks Using Resting State-fMRI
Source: PLoS One. 2015 Mar 19;10(3):e0120345. doi: 10.1371/journal.pone.0120345 (PMC4366046; doi:10.1371/journal.pone.0120345)

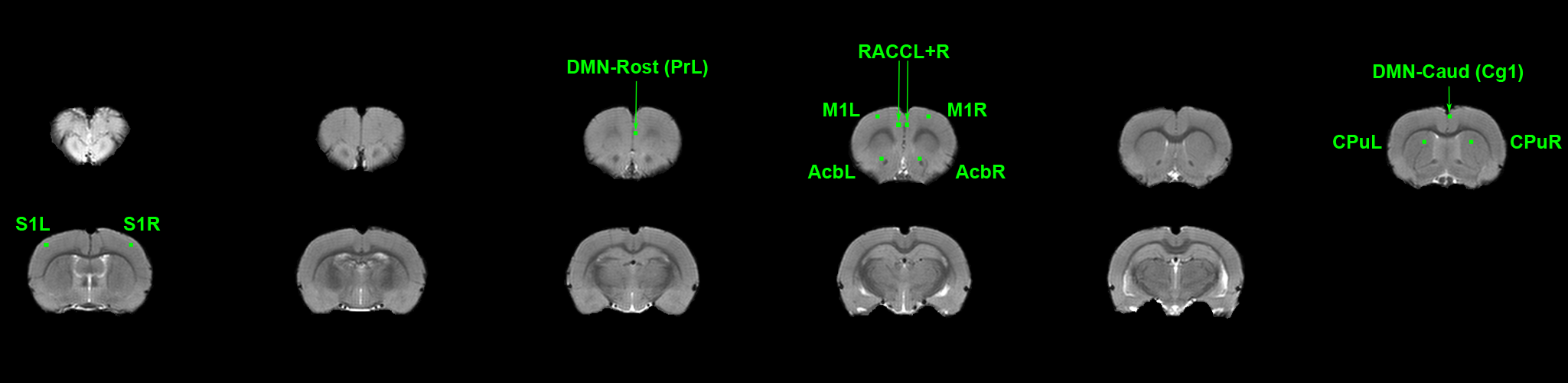

Supplement: S1 Fig — DMN-Rost = PrL; Prelimbic Cortex, RACC = Rostral Anterior Cingulate Cortex, M1 = primary motor cortex, Acb = n. Accumbens, DMN-Caud = Cg1; Cingulate Cortex area 1, CPu = Caudatus Putamen, S1 = primary somatosensory cortex. (TIF) [file pone.0120345.s001.tif]

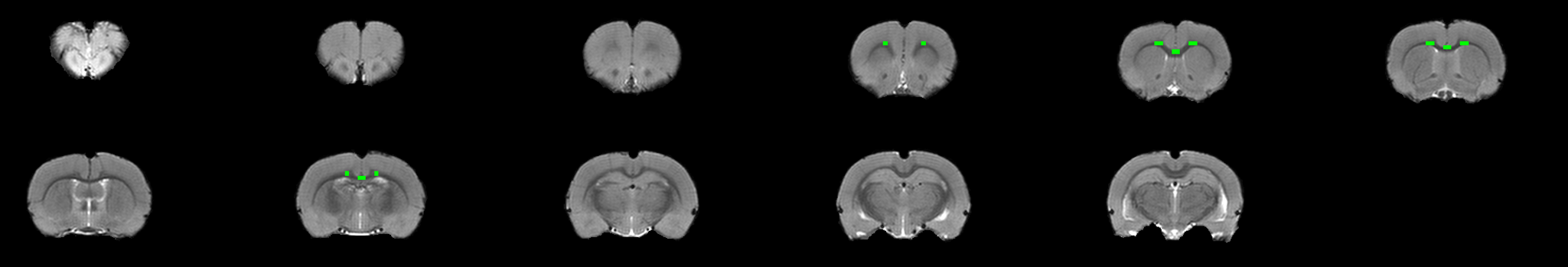

Supplement: S2 Fig — (TIF) [file pone.0120345.s002.tif]

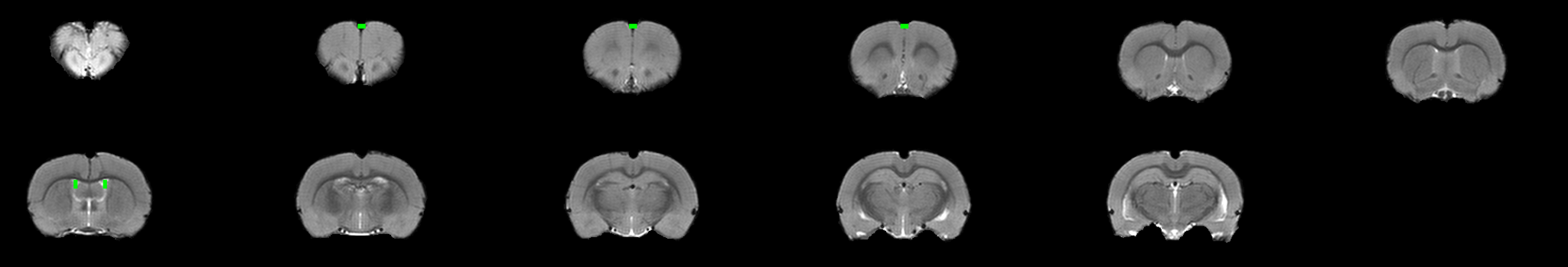

Supplement: S3 Fig — (TIF) [file pone.0120345.s003.tif]

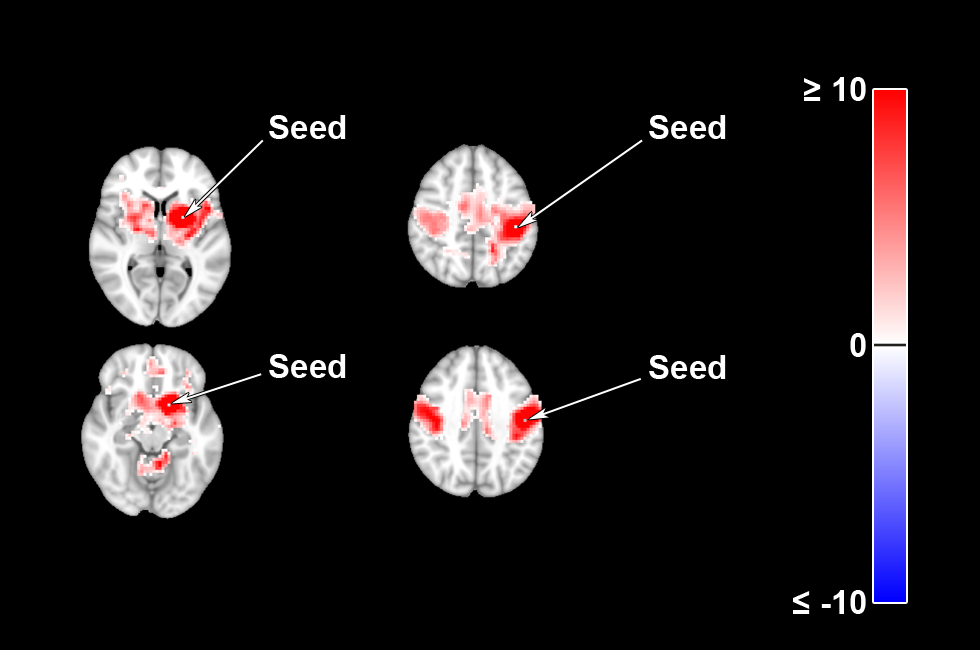

Supplement: S4 Fig — Top left = the dorsal basal ganglia network top right = the somatosensory network bottom left = the ventral basal ganglia network bottom right = the motor network. (TIF) [file pone.0120345.s004.tif]

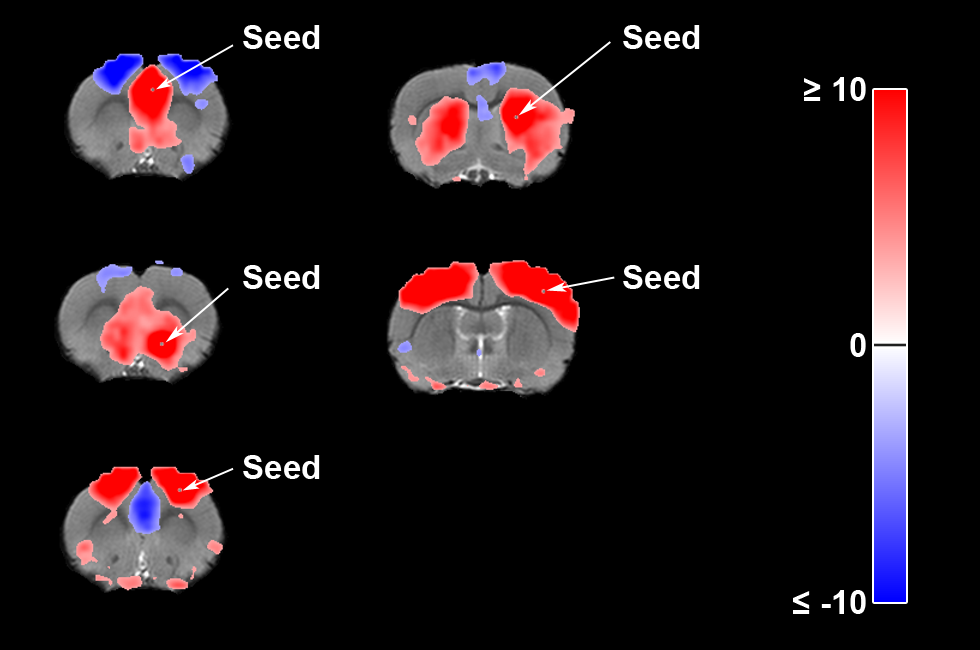

Supplement: S5 Fig — Top left = the DMN top right = dorsal basal ganglia network middle left = the ventral basal ganglia network middle right = the somatosensory network middle left = the motor network. (TIF) [file pone.0120345.s005.tif]

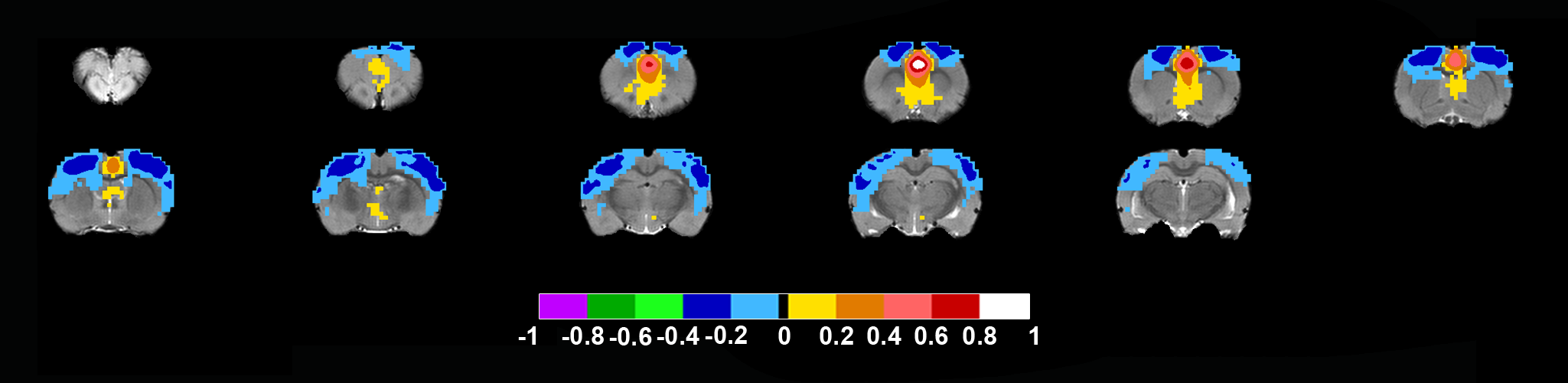

Supplement: S6 Fig — (TIF) [file pone.0120345.s006.tif]

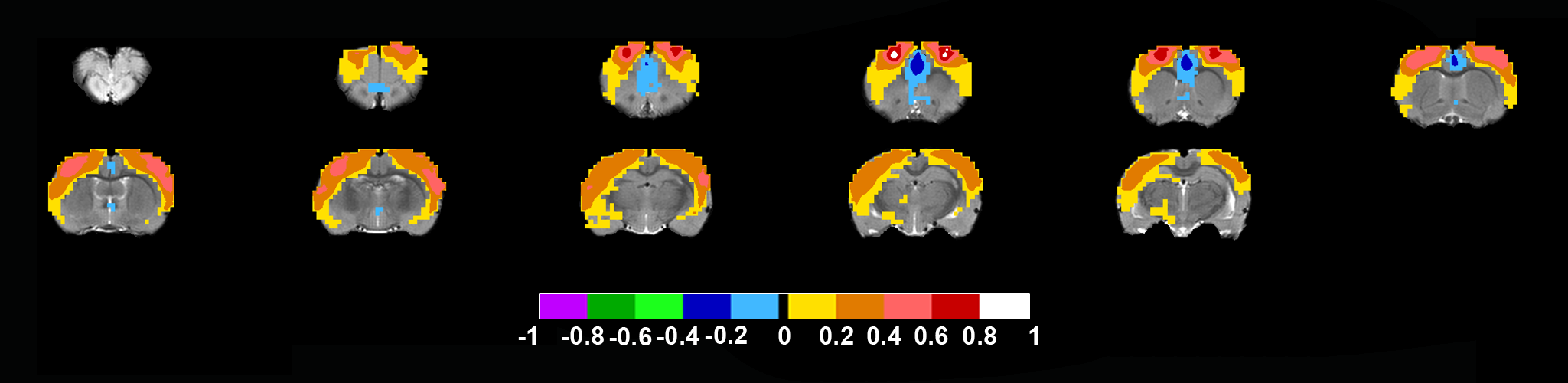

Supplement: S7 Fig — (TIF) [file pone.0120345.s007.tif]

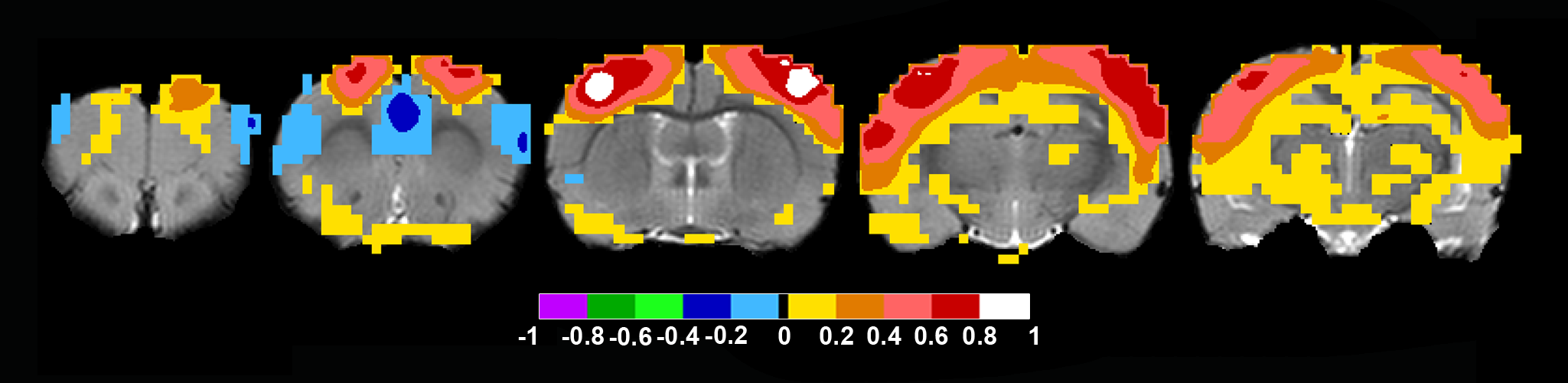

Supplement: S8 Fig — (TIF) [file pone.0120345.s008.tif]

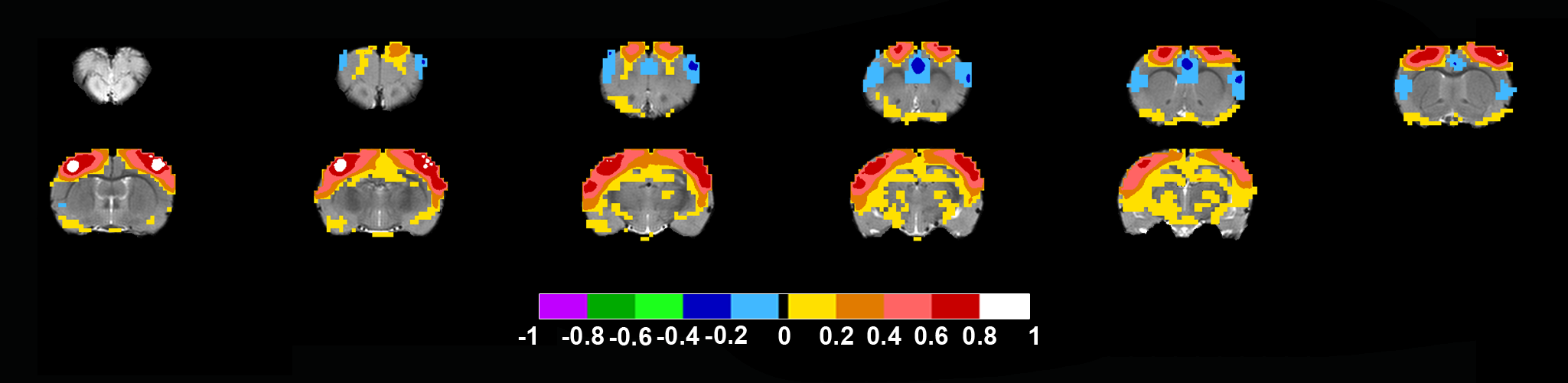

Supplement: S9 Fig — (TIF) [file pone.0120345.s009.tif]

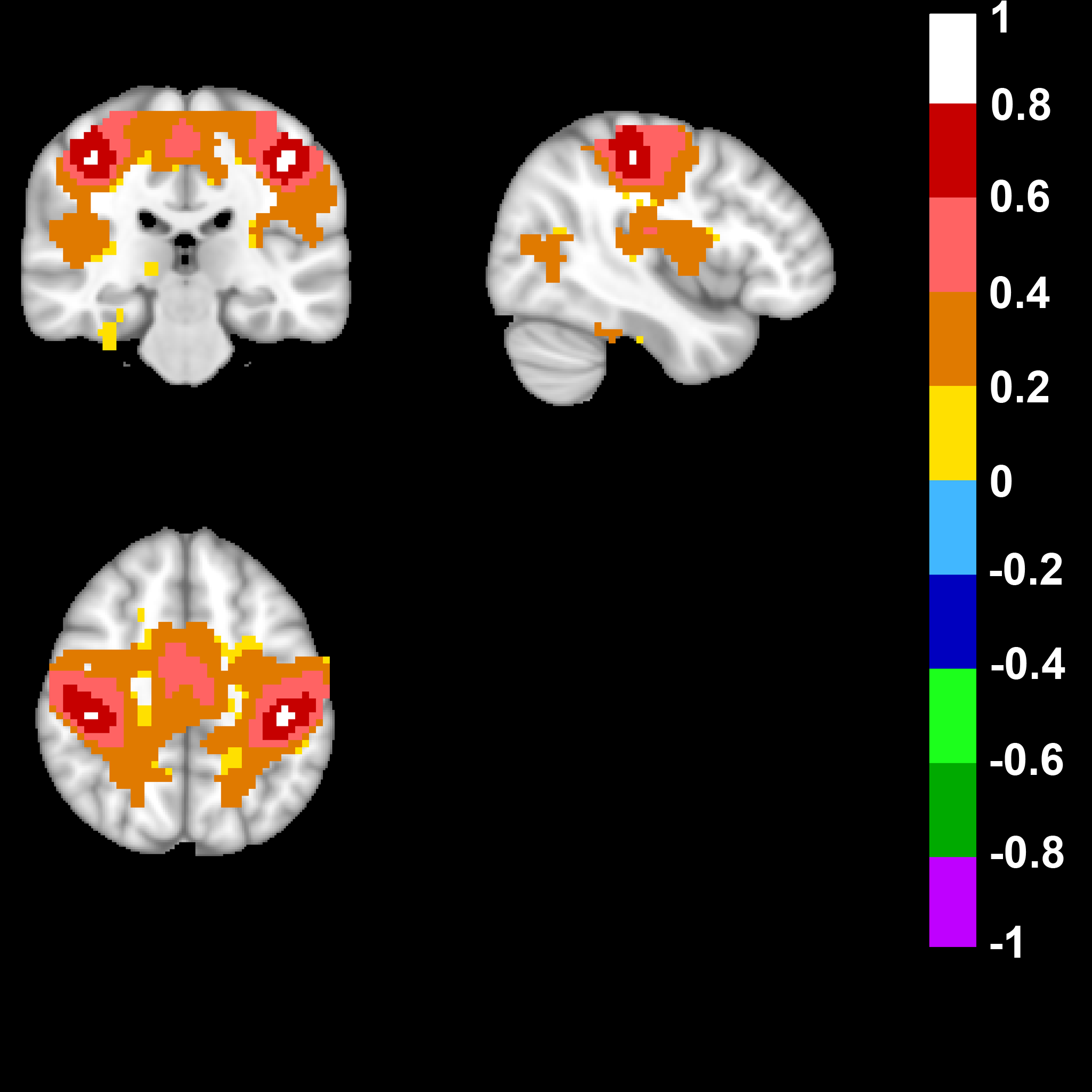

Supplement: S10 Fig — (TIF) [file pone.0120345.s010.tif]

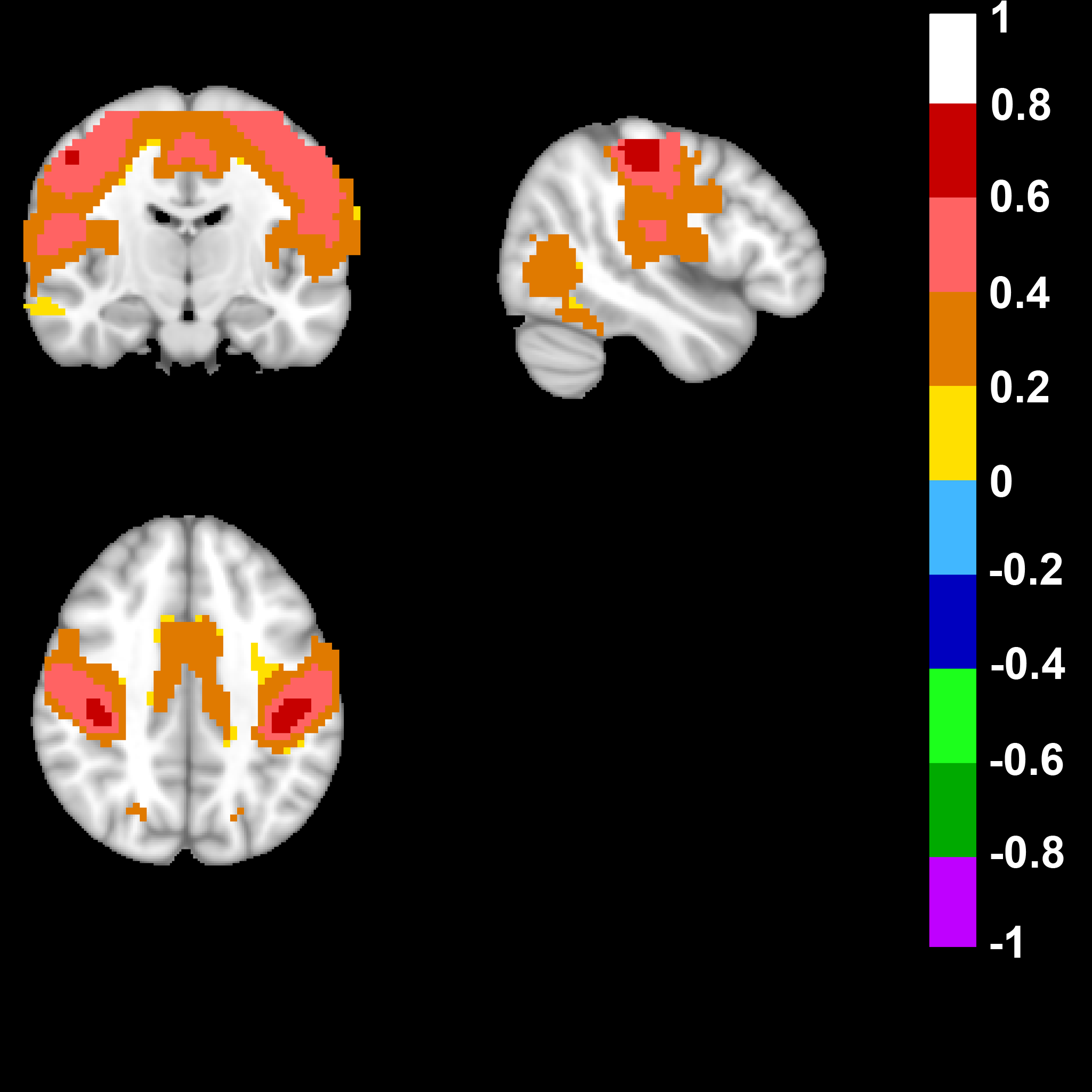

Supplement: S11 Fig — Displayed in the primary motor cortex orthogonal view. (TIF) [file pone.0120345.s011.tif]

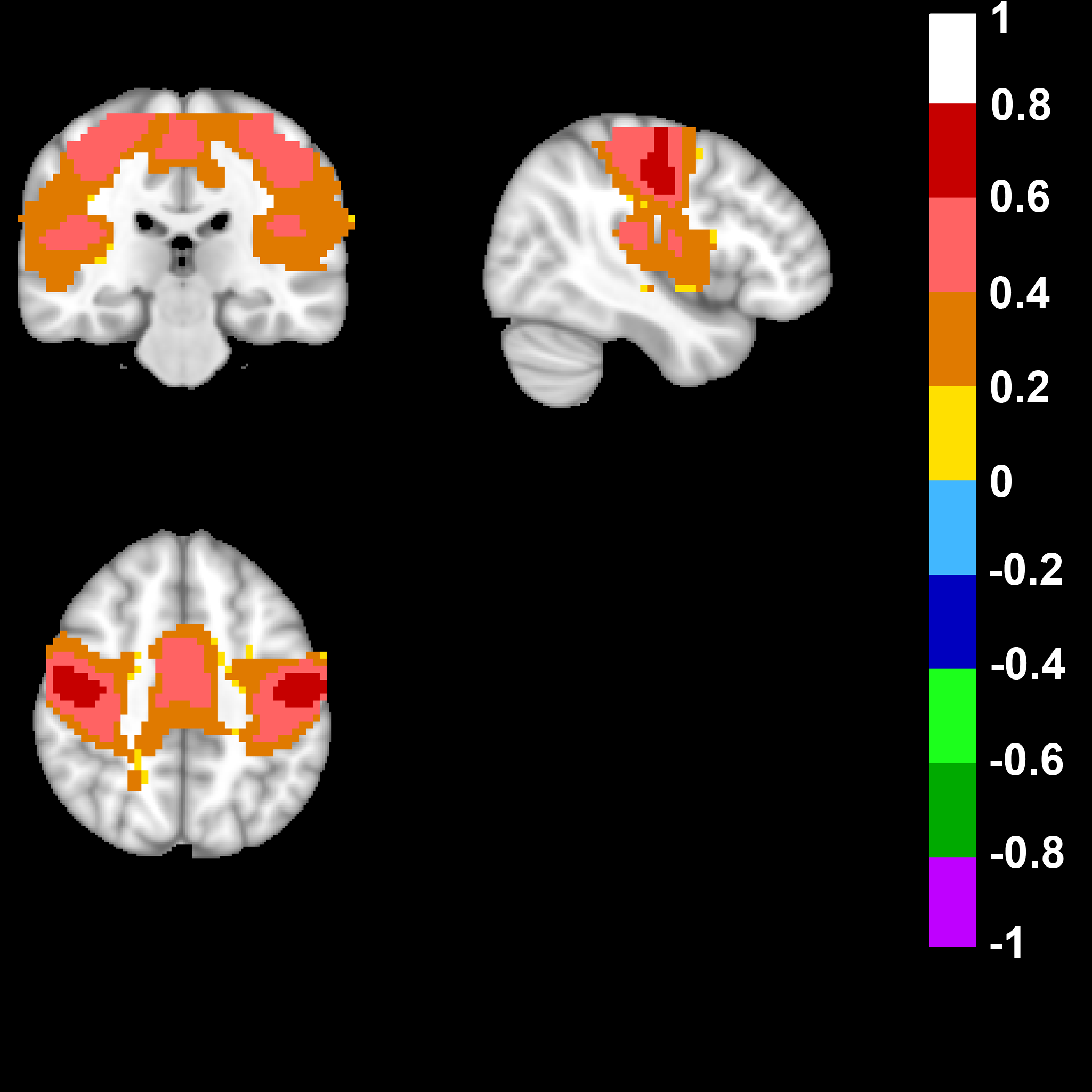

Supplement: S12 Fig — Displayed in the primary somatosensory cortex orthogonal view. (TIF) [file pone.0120345.s012.tif]

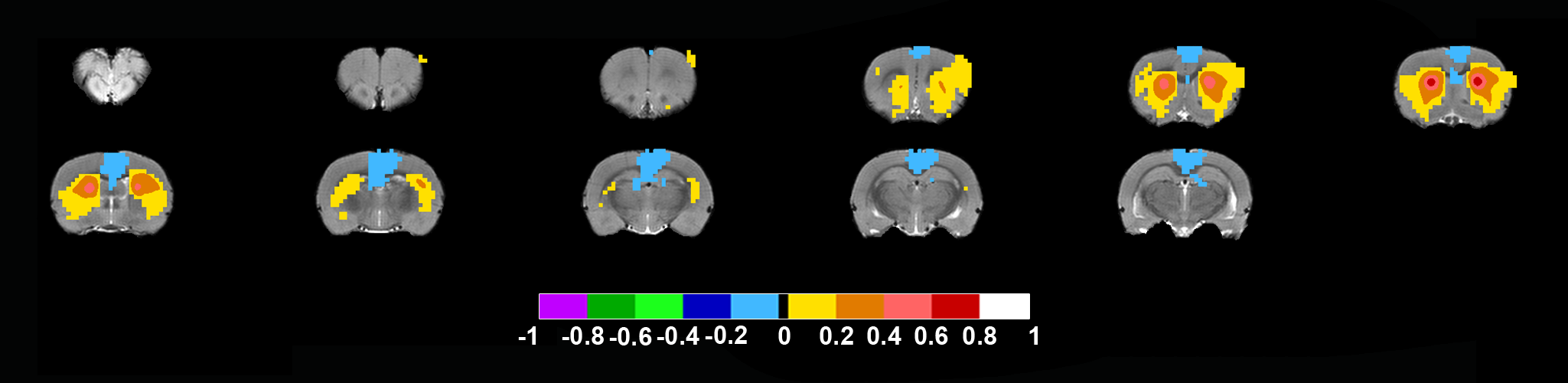

Supplement: S13 Fig — (TIF) [file pone.0120345.s013.tif]

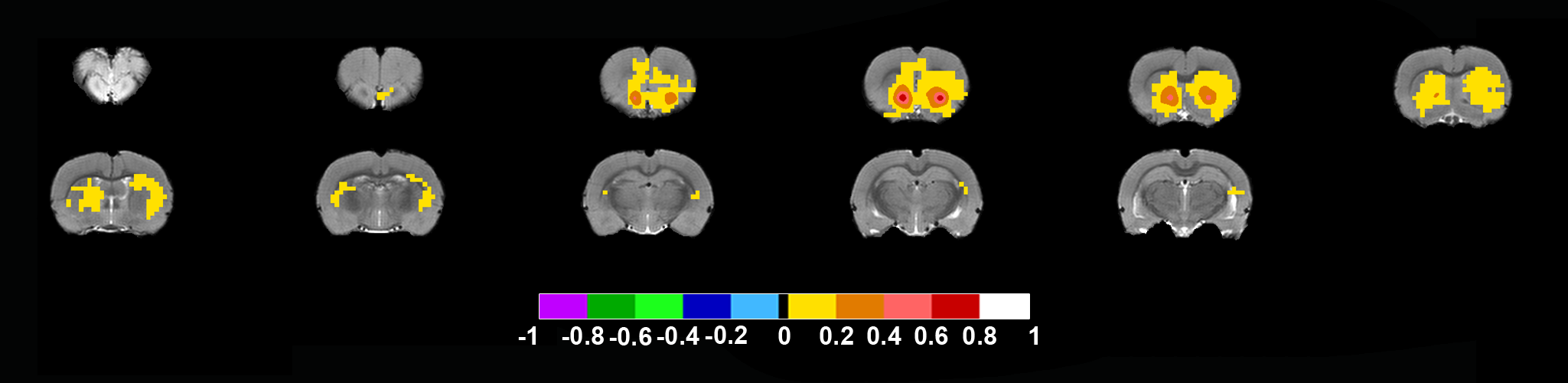

Supplement: S14 Fig — (TIF) [file pone.0120345.s014.tif]

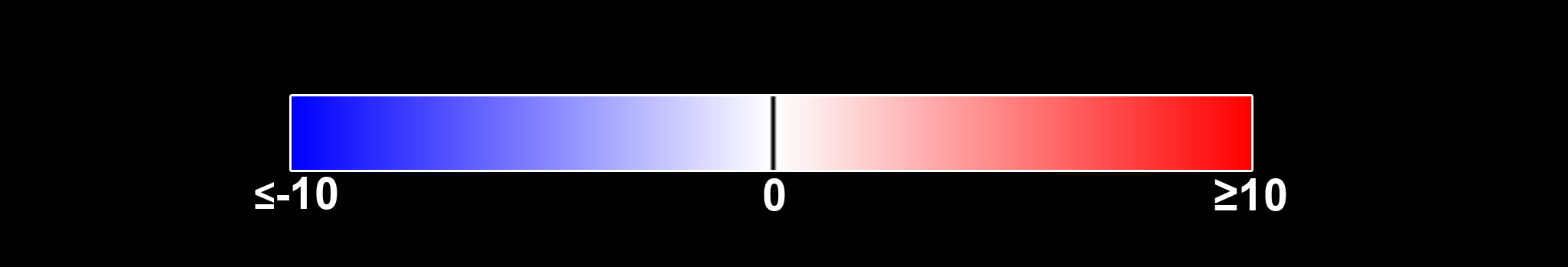

Supplement: S15 Fig — (TIF) [file pone.0120345.s015.tif]

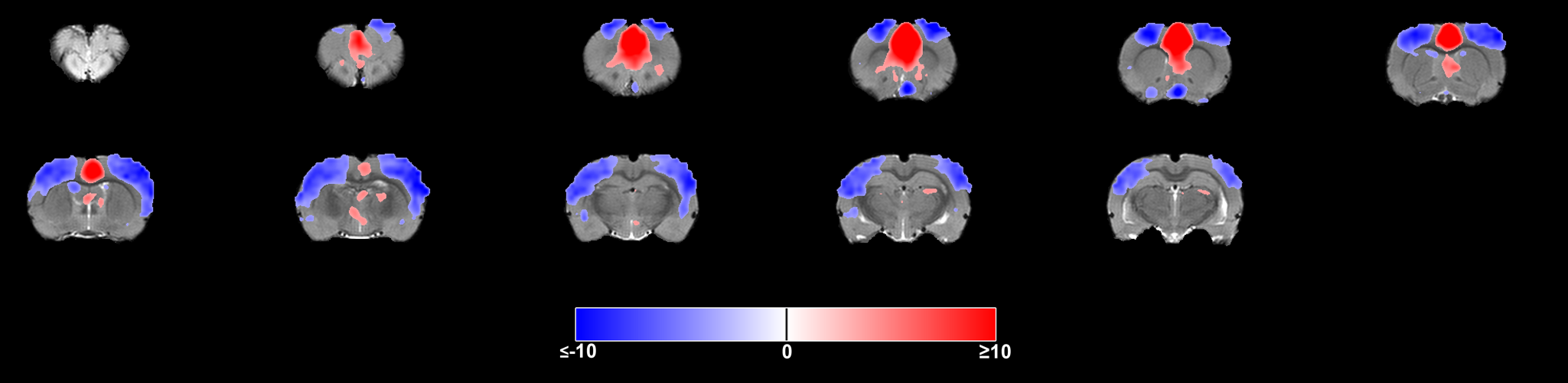

Supplement: S16 Fig — (Color scale indicates T-values) (TIF) [file pone.0120345.s016.tif]

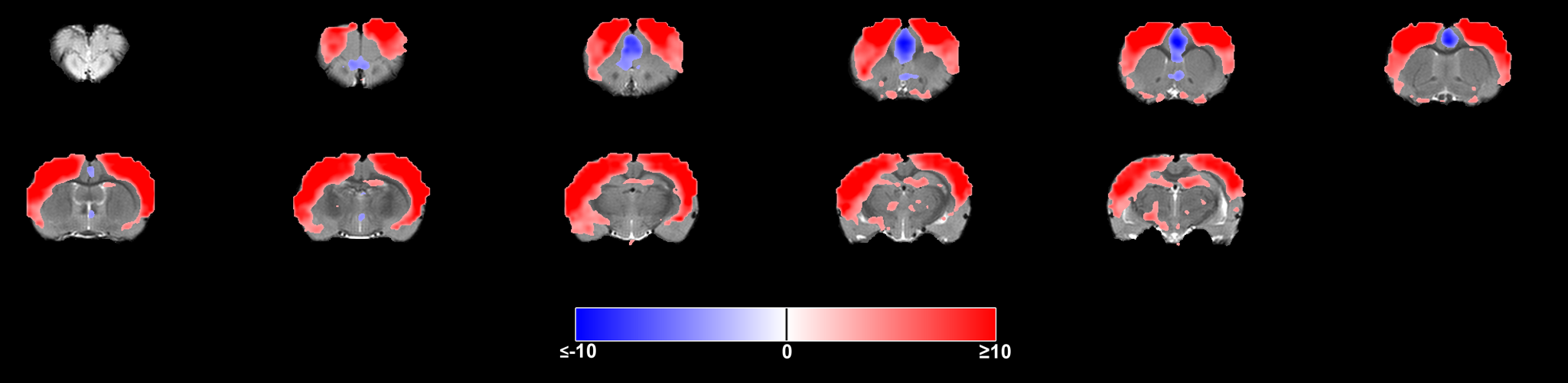

Supplement: S17 Fig — (Color scale indicates T-values) (TIF) [file pone.0120345.s017.tif]

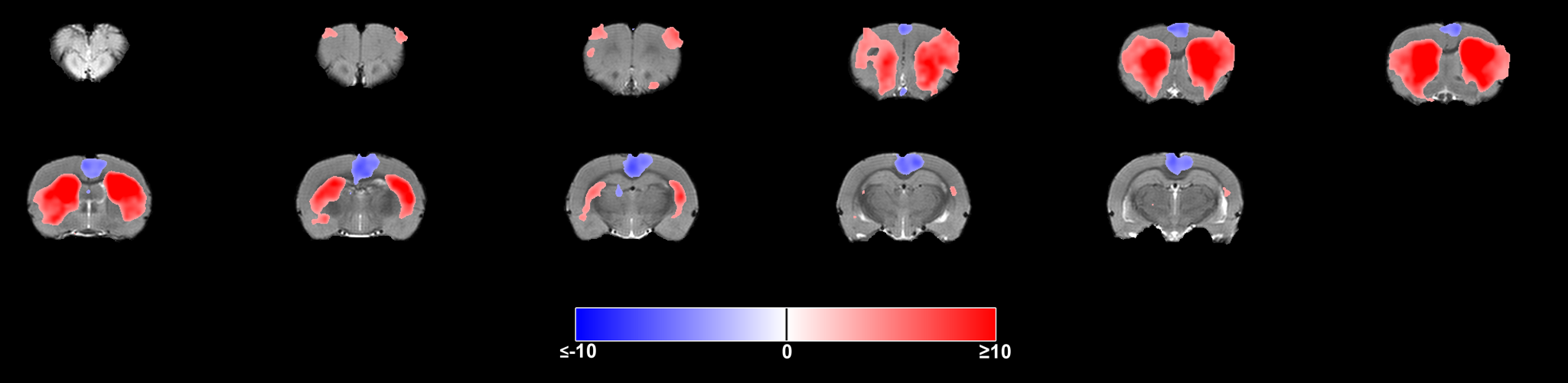

Supplement: S18 Fig — (Color scale indicates T-values) (TIF) [file pone.0120345.s018.tif]

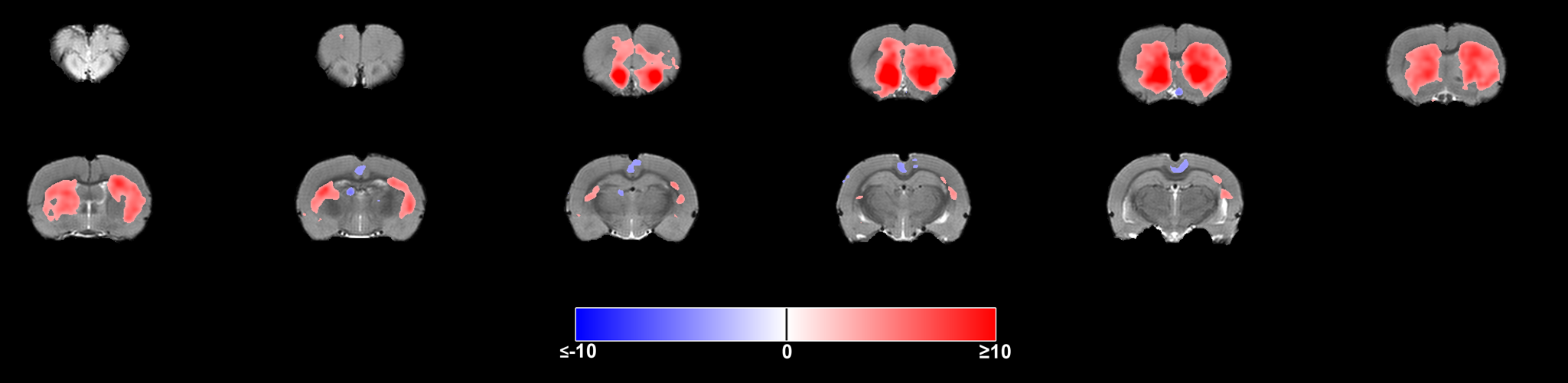

Supplement: S19 Fig — (Color scale indicates T-values) (TIF) [file pone.0120345.s019.tif]

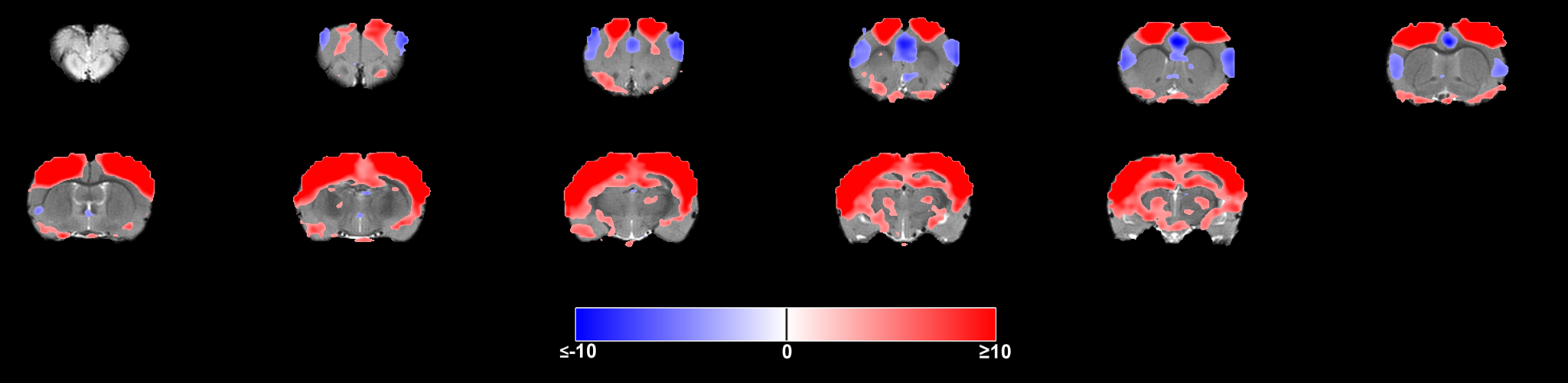

Supplement: S20 Fig — (Color scale indicates T-values) (TIF) [file pone.0120345.s020.tif]
