# Supplementary material for: Default Mode Network, Motor Network, Dorsal and Ventral Basal Ganglia Networks in the Rat Brain: Comparison to Human Networks Using Resting State-fMRI
Source: PLoS One. 2015 Mar 19;10(3):e0120345. doi: 10.1371/journal.pone.0120345 (PMC4366046; doi:10.1371/journal.pone.0120345)
Supplement: S1 Table — (DOCX) [file pone.0120345.s021.docx]

**Table S1.** **Human subject details.**

| **Subject** | **File name** | **EPI volumes** | **Age** | **Sex** |
| --- | --- | --- | --- | --- |
| Subject 1 | con01 | 180 | 26 | M |
| Subject 2 | con02 | 180 | 27 | M |
| Subject 3 | con03 | 180 | 32 | M |
| Subject 4 | con04 | 180 | 28 | M |
| Subject 5 | con05 | 200 | 27 | M |
| Subject 6 | con06 | 200 | 22 | M |
| Subject 7 | con07 | 200 | 22 | M |
| Subject 8 | con08 | 200 | 24 | M |
| Subject 9 | con09 | 200 | 27 | M |
| Subject 10 | con10 | 200 | 45 | M |
| Subject 11 | con11 | 200 | 23 | M |
| Subject 12 | con12 | 200 | 47 | M |
| Subject 13 | con13 | 200 | 35 | M |

M=male
